# Neuroprotective Effect of Combination Therapy of Glatiramer Acetate and Epigallocatechin-3-Gallate in Neuroinflammation

**DOI:** 10.1371/journal.pone.0025456

**Published:** 2011-10-13

**Authors:** Katja Herges, Jason M. Millward, Nicole Hentschel, Carmen Infante-Duarte, Orhan Aktas, Frauke Zipp

**Affiliations:** 1 Department of Neurology, University Medicine Mainz, Mainz, Germany; 2 Department of Neurology and Neurological Sciences, Stanford University, Stanford, California, United States of America; 3 Experimental and Clinical Research Center, Charité University Hospital Berlin, Berlin, Germany; 4 Department of Neurology, Heinrich-Heine-University of Düsseldorf, Düsseldorf, Germany; Centre de Recherche Public de la Santé (CRP-Santé), Luxembourg

## Abstract

Multiple sclerosis (MS) is an inflammatory autoimmune disease of the central nervous system. However, studies of MS and the animal model, experimental autoimmune encephalomyelitis (EAE), indicate that neuronal pathology is the principle cause of clinical disability. Thus, there is need to develop new therapeutic strategies that not only address immunomodulation but also neuroprotection. Here we show that the combination therapy of Glatiramer acetate (GA), an immunomodulatory MS therapeutic, and the neuroprotectant epigallocatechin-3-gallate (EGCG), the main phenol in green tea, have synergistic protective effects *in vitro* and in the EAE model. EGCG and GA together led to increased protection from glutamate- and TRAIL-induced neuronal cell death *in vitro*. EGCG combined with GA induced regeneration of hippocampal axons in an outgrowth assay. The combined application of EGCG and GA did not result in unexpected adverse events *in vivo*. Neuroprotective and neuroregenerative effects could be translated in the *in vivo* model, where combination treatment with EGCG and GA significantly delayed disease onset, strongly reduced clinical severity, even after onset of symptoms and reduced inflammatory infiltrates. These results illustrate the promise of combining neuroprotective and anti-inflammatory treatments and strengthen the prospects of EGCG as an adjunct therapy for neuroinflammatory and neurodegenerative diseases.

## Introduction

Multiple Sclerosis (MS) is a common inflammatory autoimmune disease of the central nervous system (CNS) in which myelin-specific CD4+Th1 and Th17 cells are thought to orchestrate effector processes resulting in the destruction of the myelin sheath [1]. Recent studies in MS and the animal model, experimental autoimmune encephalomyelitis (EAE) suggest that already during the early phase of disease, neuronal pathology involving axonal transection and loss of parental cell bodies play a pivotal role in clinical severity and correlate better with long-term disability than the degree of demyelination [2], [3], [4]. Regarding the underlying mechanisms, we showed that tumor necrosis factor related apoptosis inducing ligand (TRAIL) contributes to inflammatory neurodegeneration [5]. Activated T cells and microglia secrete TRAIL, which acts as a death signal on neurons and susceptible oligodendrocytes [5], [6], [7]. Furthermore, excitotoxicity and oxidative stress play a major role as mediators of axonal damage [8]. However, currently approved treatments focus primarily on immunomodulation, and are only partially effective. Thus, new therapeutic strategies that include neuroprotective as well as neuroregenerative aspects are required. One promising strategy is the use of combination therapies based on drugs that may complement one another in order to provide additive benefit [9].

Glatiramer acetate (GA, Copaxone, Copolymer 1) is an immunomodulatory agent for treatment of relapsing-remitting MS. GA is a synthetic basic random copolymer composed of tyrosine, glutamate, alanine and lysine that induces GA-specific Th2 cells which secrete anti-inflammatory cytokines in the CNS through cross-recognition with myelin autoantigens [10]. Recently, it has been shown that GA promotes neurogenesis, neuroprotection and remyelination in MS lesions mediated by neurotrophic factors such as brain-derived neurotrophic factor (BDNF), Neurotrophin 3 and Neurotrophin 4 produced by GA-specific Th2 cells [11], [12], [13]. New studies also indicate that GA can induce anti-inflammatory type II monocytes, that are characterized by increased secretion of interleukin (IL)-10 and decreased production of IL-12 and that can direct differentiation of Th2 cells and CD4+CD25+FoxP3+ regulatory T cells [14].

We have recently shown that epigallocatechin-3-gallate (EGCG), the main phenol of green tea, can reduce clinical severity of EAE by both limiting brain inflammation and reducing neuronal damage [15]. EGCG is capable of protecting against neuronal damage by directly blocking the formation of reactive oxygen species in neurons as well as through iron chelating and anti-apoptotic functions [15], [16], [17].

In light of the immunomodulatory and neuroprotective properties of GA and EGCG we hypothesized an additive or synergistic protective effect of GA and EGCG in inflammation as well as in neuroprotection *in vitro* and *in vivo* in the animal model of MS. Moreover, we wanted to know whether the combined application of the two substances was safe, in order to be able to start a treatment trial in patients.

Combination therapy of GA and EGCG synergistically reduced neuronal cell death and promoted axonal outgrowth of primary neurons. These effects could be translated into the EAE model in which diminished clinical disease severity was associated with reduced CNS inflammation in a synergistic manner. These results strengthen the prospects of EGCG as an adjunct and well-tolerated therapy for neuroinflammatory diseases, and underscore the importance of evaluating combined anti-degenerative and anti-inflammatory treatments.

## Materials and Methods

### Ethical Considerations

Animals were housed in the animal facility of the Neuroscience Research Center of the Charité Universitätsmedizin Berlin. Experimental procedures were approved by the regional animal study committee of Berlin (LAGeSo approval ID G0147/05). All animal work was conducted in accordance to national and international guidelines to minimize discomfort to animals.

### CNS cell death assays

For assessment of viability, hippocampal HT22 cells were seeded at 5,000 cells per well in 96 well plates and cultured for 24 h. On the following day, cells received fresh medium and were incubated with 10 µg/ml EGCG, 50 µg/ml GA, or both compounds for 2 h before incubation with 5 mM glutamate. In parallel, glioblastoma LN18 cells were cultured at 30,000 cells per well along with 10 µg/ml EGCG and/or 12.5 µg/ml GA, and cell death was induced by incubation with TRAIL (20 ng/ml, Alexis, San Diego, CA) and TRAIL enhancer (2 µg/ml). The crystal violet assay was performed 16 h thereafter as previously described [15]. Briefly, cells were stained for 30 min with 0.5% crystal violet in 20% methanol. After washing and drying overnight, crystal violet was dissolved in 50 µl/well of 0.1 M sodium citrate solution diluted in 50% ethanol and subsequently quantified photometrically by absorbance at 550 nm (Dynatech Laboratories, Chantilly, VA). Values are expressed as the percentage of survival compared with untreated controls.

### Axonal outgrowth assay

Entorhinal cortex slices were prepared at postnatal day 2 from mouse brains as previously described [18], [19]. In brief, after decapitation the entorhinal cortex was dissected in ice-cold preparation medium (minimal essential medium with 2 mM L-glutamine and 8 mM Trisbase) [20]. Transverse slices of 350 µm were cut using a tissue chopper. Entorhinal slices were embedded in 30 µl of collagen drops and placed on a glass slide. The preparations were incubated with 5 µg/ml EGCG, 6.25 µg/ml GA, or both compounds together. To evaluate axonal outgrowth from the explants we used a protocol utilizing image analysis software (Image J, Wayne Rasband, NIH, as described previously [21], [22]. In brief, axonal density and number of axons were quantified after 2 days of culture, and photo-documented at a total magnification of 100×, Olympus LCPLANFL objective (Olympus IX70, Hamburg, Germany) [23]. Image processing was based on an edge detection algorithm, the Sobel operator, which performs a 2-dimensional spatial gradient measurement in a microphotograph to detect regions of high spatial density that correspond to neurites. To determine the axonal density, the mean intensity was calculated in a standardized area parallel to the brain slice margin of every single brain slice [23]. The number of axons was measured by quantifying all intersections of axons with a standardized line parallel to the brain slice border. Supernatants were reserved from the slice cultures and analyzed for neurotrophins by Enzyme-Linked Immunosorbent Assay (ELISA), according to the manufacturer's directions (Promega, Mannheim Germany). The limit of detection was 31.2 and 15.6 pg/ml for glial cell derived neurotrophic factor (GDNF) and BDNF, respectively.

### Induction and treatment of EAE

6–8 week old female SJL/L mice (Charles River Laboratories, Sulzfeld, Germany) were immunized s.c. with 250 µg of proteolipid protein (PLP) peptide 139–151 (purity >95%; Pepceuticals, Leicester, UK) emulsified in an equal volume of PBS and complete Freund's adjuvant containing 6 mg/ml Mycobacterium tuberculosis H37Ra (Difco, Franklin Lakes, NJ). 200 ng of Bordetella pertussis toxin (List Biological Laboratories, Campell, CA) was administered i.p. at day 0 and 2. For preventive treatment, EGCG (Sigma-Aldrich, Deisenhofen, Germany) was dissolved in 0.9% NaCl. 300 µg of EGCG or vehicle (0.9% NaCl) were given by oral gavage twice daily from day-9 before immunization on. 50 µg GA (TEVA) or vehicle (Mannitol 4%) was dissolved in PBS, emulsified in incomplete Freund's adjuvant (Difco) and injected s.c. once on day −7. Therapeutic treatment with 300 µg of EGCG or vehicle twice daily, and with 150 µg GA or vehicle once daily, was started after animals reached a clinical score≥2.

Mice were monitored daily for clinical symptoms and scored as follows: 0 = no disease; 1 = complete tail paralysis; 2 = hind limb paraparesis; 3 = bilateral hind limb paraplegia; 4 = complete forelimb and hind limb paraplegia; 5 = moribund or dead animals.

### Histology

Spinal cords from mice transcardially perfused with 4% paraformaldehyde were postfixed overnight, cryoprotected successively in 15% and 30% sucrose and cut into 8 cross-sectional segments. The segments were embedded together in Cryo-Embed (Ax-lab), frozen in a methylbutane/dry ice mixture and cut into 12 µm sections with a cryostat. Hematoxylin and eosin stained sections were examined by light microscopy for the presence of inflammation, using a Zeiss Observer.Z1 microscope with a Zeiss AxioCam MRm camera. Semi-quantitative assessment was done by counting the number of quadrants with inflammatory infiltrates for each of the 8 segments. Quadruplicate sections for each mouse were assessed in a blinded manner and data are presented as the percentage of total quadrants positive for inflammatory infiltrates.

### Statistics

EAE disease courses, histology scores, and cumulative disease activity were analyzed with the non-parametric Kruskal-Wallace ANOVA with the Dunn's post-hoc test, and the Mann-Whitney test. The cell death and axonal outgrowth assays were analyzed with 1-way ANOVA with the Bonferroni correction. Time to disease onset was analyzed with the logrank test, using the Bonferroni correction for multiple comparisons. GraphPad Prism 4 (GraphPad Software, San Diego CA) was used for the analysis. p-values<0.05 were considered significant (* p<0.05; ** p<0.01; *** p<0.001).

## Results

### EGCG and GA in combination inhibit death of CNS cells *in vitro*

We asked whether the combination of EGCG and GA could have an additive or synergistic effect in preventing neuronal damage in mouse and human CNS cell lines. Therefore, we induced neuronal cell death through two mechanisms, which are known to contribute to neuronal damage in neuroinflammation, glutamate-promoted excitotoxicity and TRAIL-mediated apoptosis.

**Figure 1:**
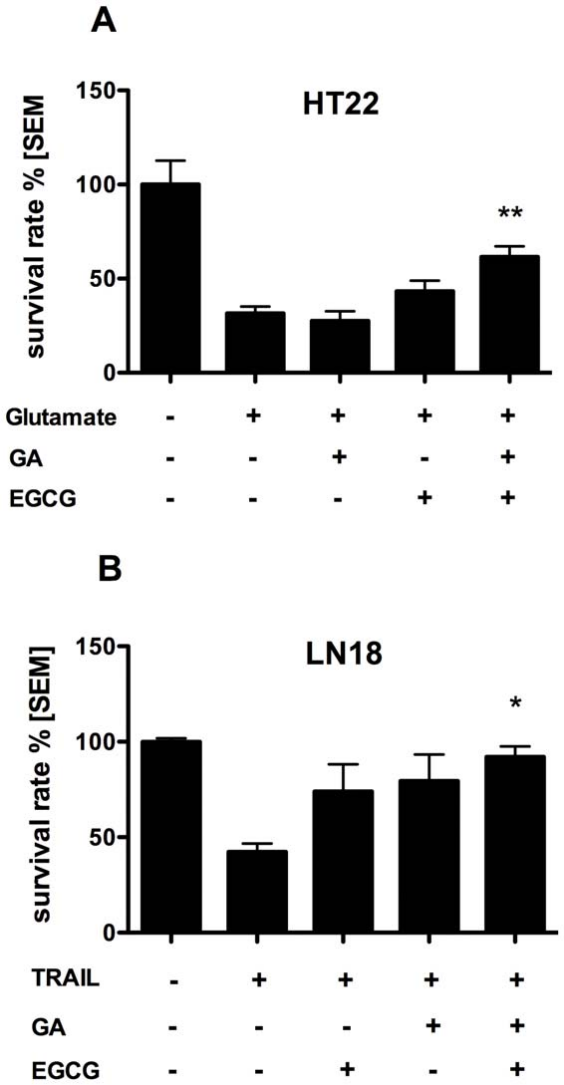
EGCG and GA in combination inhibit death of CNS cells *in vitro*. Survival of murine HT22 neuronal cells (A) and human glioblastomal LN18 cells (B) was determined in a crystal violet assay after induction of apoptosis by incubation with Glutamate (5 mM) (A) or TRAIL (20 ng/ml) (B). EGCG (10 µg/ml) and/or GA (12.5 µg/ml) were added to the culture 2 h before apoptosis induction, *p<0.05, **p<0.001, representative of 2 experiments.

We induced excitotoxicity by incubation of murine hippocampal HT22 cells with glutamate. We preincubated the cells with different concentrations of EGCG and GA and established suboptimal doses of EGCG (10 µg/ml) or GA (50 µg/ml). Suboptimal doses of EGCG and GA showed little or no neuroprotective effect compared to control, whereas the combination of 10 µg/ml EGCG and 50 µg/ml GA significantly increased survival of HT22 cells compared to controls, as determined by the crystal violet assay (Fig. 1A). Thus, the effect of the combination therapy was found to be synergistic compared to EGCG and GA alone.

To examine whether the combination therapy is also protective in a caspase-dependent apoptotic pathway, we induced TRAIL-mediated cell death in susceptible human glioblastomal LN18 cells. Again, the combination of EGCG (10 µg/ml) and GA (12.5 µg/ml) showed a statistically significant increase in cell survival compared to control. Conversely, EGCG or GA alone did not induce a significant increase (Fig. 1B).

### EGCG and GA promote axonal growth and density *in vitro*

**Figure 2:**
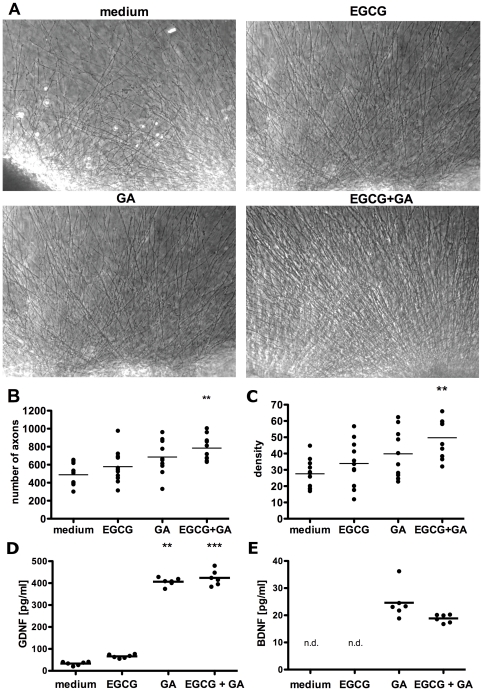
EGCG and GA promote axonal growth and density *in vitro*. Representative photomicrographs of brain slices with axonal outgrowth after addition of EGCG (5 µg/ml), GA (6.25 µg/ml) and the combination of EGCG (5 µg/ml) and GA (6.25 µg/ml) are shown. 100× magnification, scale bar: 300 µm (A). The number of axons was measured by quantifying all intersections of axons with a standardized line parallel to the brain slice border, **p<0.001 (B). The density of outgrowing axons was determined by measuring the mean intensity in a standardized area parallel to the brain slice edge, **p<0.001 (C). Levels of GDNF (D) and BDNF (E) were measured in the supernatant of outgrowth assays by ELISA, n.d. = not detectable. **p<0.001 ***p<0.0001.

We next investigated whether EGCG and GA stimulate neuroregeneration using an axonal outgrowth model of entorhinal cortex explants. The explants were embedded in a three-dimensional collagen gel matrix with 5 µg/ml EGCG or 6.25 µg/ml GA alone or with the combination of 5 µg/ml EGCG and 6.25 µg/ml GA. The density of the outgrowing neurites was photo-documented at higher magnification and quantified (Fig. 2A). Axonal density was quantified by measuring the mean intensity in a standardized area parallel to the brain slice margin.

The total number of axons increased significantly in the presence of EGCG and GA in combination compared to control brain slices (Fig. 2B). GA or EGCG alone did not induce a significant increase in the number of neurites.

Axons in slices treated with both GA and EGCG showed a significantly higher density compared to brain slices under control conditions. Again, there was no significant effect of EGCG or GA alone (Fig. 2C). Thus, the combination of EGCG and GA showed a synergistic effect on axonal growth and density compared to EGCG and GA alone. In addition, GA induced the secretion of BDNF and GDNF, which was detected in the supernatants of the brain slice culture by ELISA. No additional increase was seen with EGCG (Fig. 2D, E).

### Combination therapy with EGCG and GA delays disease onset and synergistically reduces EAE severity

Next, we tested whether these neuroprotective and neuroregenerative effects of EGCG and GA *in vitro* could translate into an additive or synergistic clinical benefit in the animal model of autoimmune neuroinflammation, EAE. In order to evaluate combination therapy in EAE using medications that are individually effective, it is necessary to test these drugs in combination using suboptimal doses of at least one drug. Therefore, SJL/L mice with EAE were treated with known suboptimal doses of GA (50 µg) supplemented with 600 µg EGCG [24].

**Figure 3:**
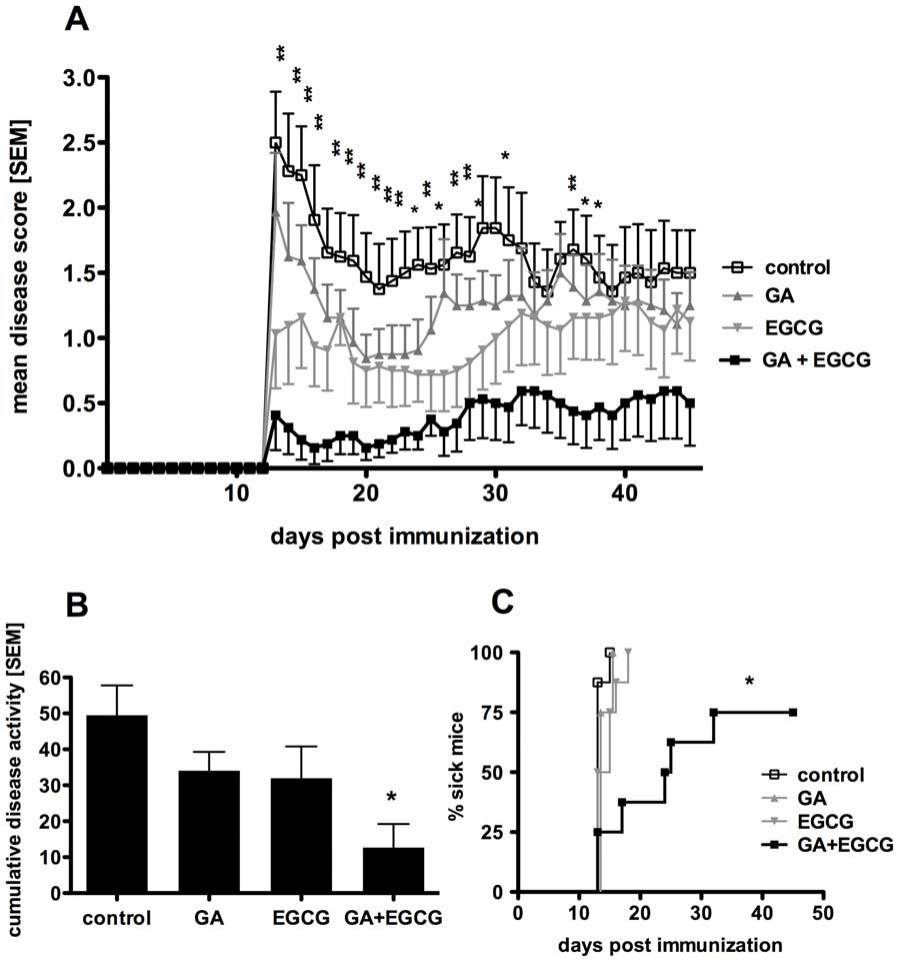
Combination therapy with EGCG and GA delays disease onset and synergistically reduces EAE severity. For preventive treatment EGCG (300 µg) or vehicle (NaCl 0.9%) was administered orally twice daily from day −9 before immunization with PLP_139–151_ until day 45. GA (50 µg) or vehicle (Mannitol 4%) in incomplete Freund's adjuvant was given s.c. once on day −7 before immunization. Mean disease score of control, EGCG, GA and the combination therapy group is shown from day 0 to day 45 after immunization (A), * p<0.05 day 13,15–18,20–22,24,26–27, **p<0.01 day 14,19,23,25,28,35–39,42, n = 8 per group. Onset of disease in control, GA, EGCG and combination therapy group is presented in a Kaplan-Meier survival curve as percentage of sick mice at a certain day after immunization (B), *p<0.05. Cumulative disease activity of control, GA, EGCG and combination therapy group was calculated as the area under the curve of the clinical score plots for each individual animal (C), *p<0.05.

For preventive treatment, EGCG was given orally twice daily starting nine days before EAE induction, and GA was injected s.c. in incomplete Freund's adjuvant once seven days before immunization with PLP_139–151_. As expected, treatment with suboptimal doses of GA alone did not significantly decrease disease incidence and severity. However, the combination therapy significantly reduced the severity of relapsing-remitting EAE and synergistically delayed the onset of disease compared to vehicle treated animals (Fig. 3A,B). The treatment effect of the combination therapy was also found to be synergistic compared to EGCG and GA treatment alone on 22 out of 33 days of disease observation. The cumulative disease activity (calculated as the area under the curve of the clinical score plots for each individual animal [25]), was significantly lower in the combined EGCG and GA group, as compared to the vehicle treated, and appeared synergistic compared to the single treatment groups (Fig. 3C). Additionally, all animals were closely monitored for adverse clinical events, such as rapid worsening of symptoms or anaphylaxis, or for signs of atypical EAE (ie. ataxia), although none of these events occurred.

### Combination therapy with EGCG and GA alleviates disability in established EAE

**Figure 4:**
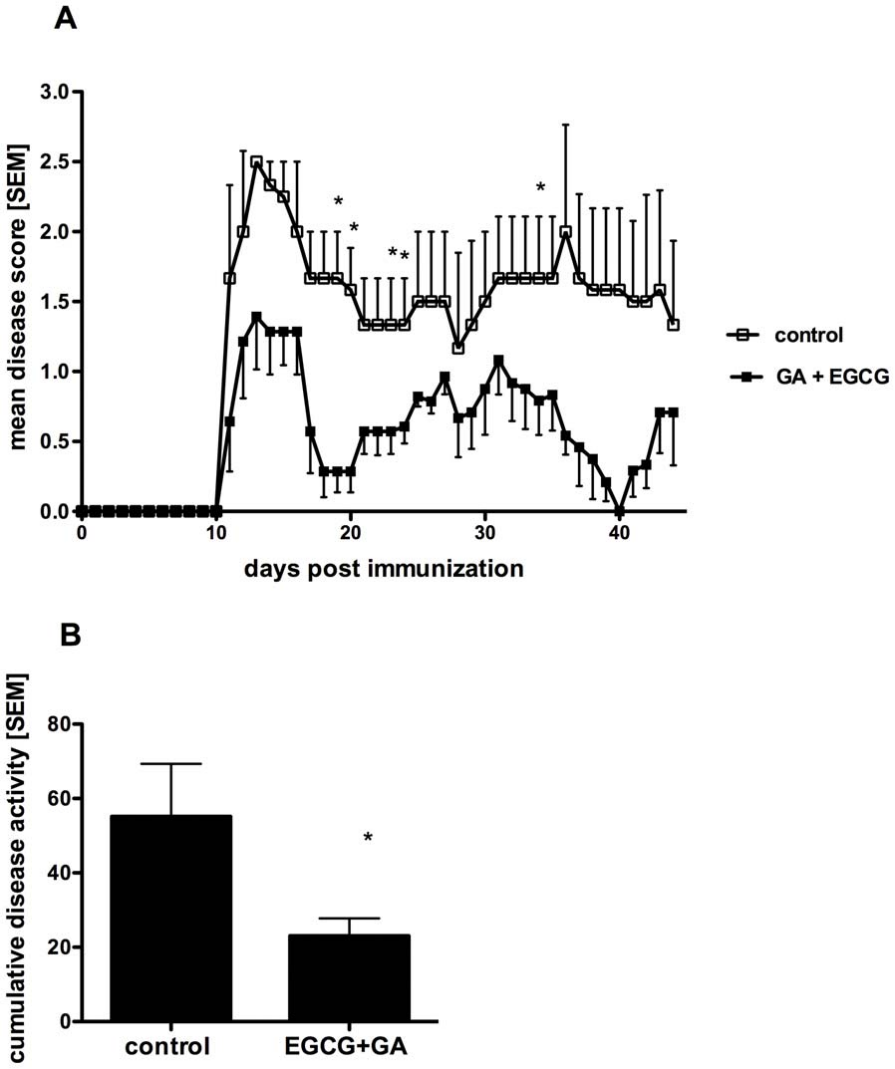
Combination therapy with EGCG and GA alleviates disability in established EAE. For treatment of acute disease PLP_139–151_-induced EAE mice were randomized into two groups after reaching a disease score of 2 and subsequently received 300 µg of oral EGCG or vehicle (0.9% NaCl) twice daily in addition to daily s.c. injections of 150 µg GA or vehicle (4% Mannitol). Mean disease score of control and combination therapy group is shown from day 0 to day 44 after immunization (A), *p<0.05 day 19, 20, 23, 24, 34, n = 6. Cumulative disease activity of control and combination therapy group was calculated (B), *p<0.05.

For the treatment of patients suffering from MS it is important to know whether the combined treatment could have a protective effect in established disease. For this therapeutic paradigm, treatment was initiated when all mice showed a clinical score≥2. EGCG (300 µg) was given twice daily, and GA (150 µg) was injected subcutaneously daily. The combination therapy enhanced recovery and prevented long-term neurological deficits, as indicated by significant reductions in the mean clinical score (Fig. 4A) and the cumulative disease activity compared to the vehicle treated group (Fig. 4B). Again, no adverse events occurred in the combination therapy group during the observation period.

### EGCG and GA together ameliorate CNS inflammatory pathology

**Figure 5:**
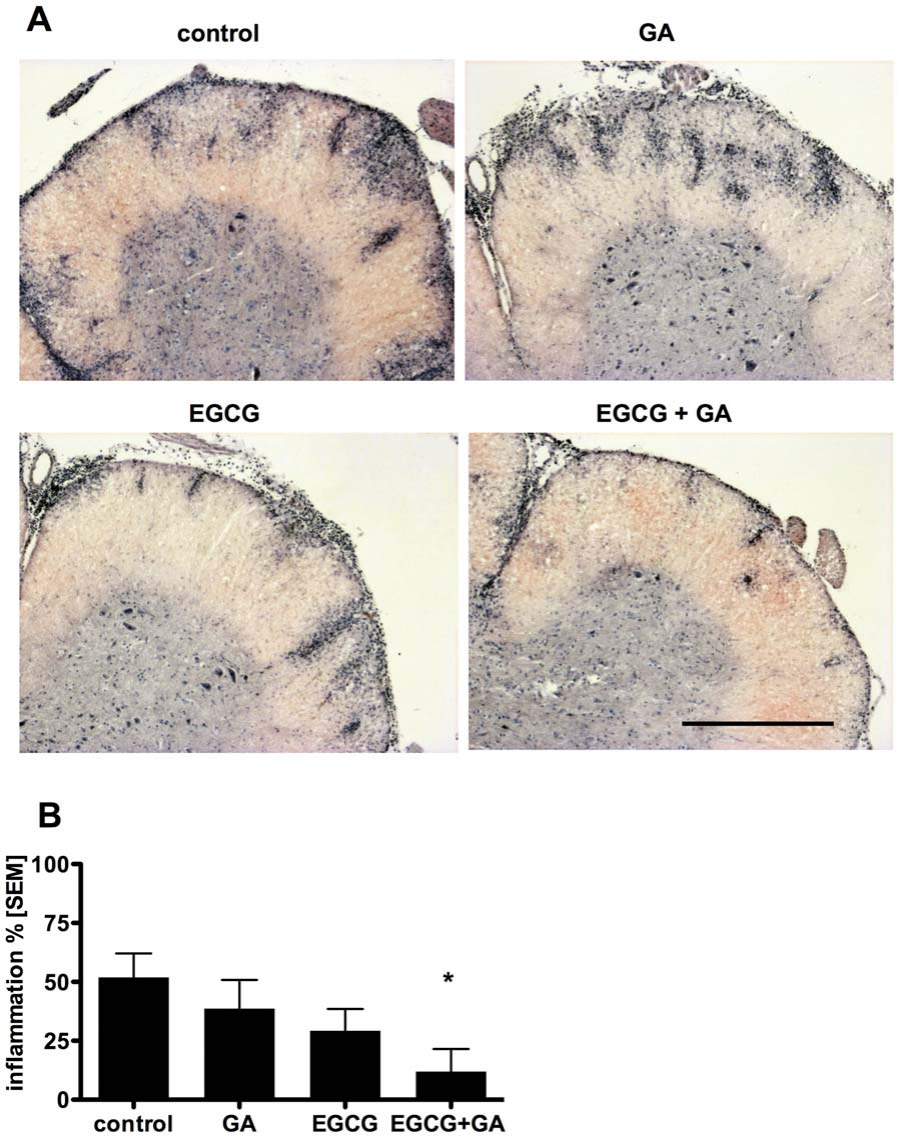
EGCG and GA together ameliorate CNS inflammatory pathology. Representative Hematoxylin and Eosin staining of transverse spinal cord sections of vehicle, EGCG, GA and EGCG and GA treated EAE mice is shown (A). 100× magnification, scale bar 500 µm. Inflammation in standardized areas of the spinal cord of all treatment groups was assessed and is presented semi-quantitatively as percentage of spinal cord quadrants with inflammatory foci relative to all assessed quadrants (B), *p<0.05, n = 8 per group.

We then examined whether the synergistic protective effects of the preventive EGCG and GA treatment on EAE clinical scores would be reflected in the CNS inflammatory pathology. Histological examination revealed extensive inflammatory lesions throughout the spinal cord in control mice. While some reduction in the histopathology could be seen in both the EGCG and GA single treated groups, this effect was synergistic in the combination treatment group (Fig. 5A). Upon quantification of the extent of inflammation, only the combined EGCG and GA treated group showed a statistically significant reduction in histopathology, compared to the control (Fig. 5B).

## Discussion

GA is one of the most common immunomodulatory therapies for MS. In addition to the inflammatory components of the pathology, neurodegeneration, axonal damage and deficits in neuroregeneration are important aspects of the disease and should be taken into consideration as targets for therapeutic approaches. Thus, we investigated the potential of the combination therapy of GA and the previously tested neuroprotective agent EGCG.

We showed a synergistic effect of the combination therapy of EGCG and GA on neuronal survival and axonal growth *in vitro*. No unexpected adverse events occurred when applying the two substances together. Importantly, we also found that the combination therapy not only delayed EAE onset and reduced severity when applied to prevent disease, but was also effective in the treatment of established EAE symptoms *in vivo*.

Neuronal cell death is a major feature in the pathology of neuroinflammatory diseases such as MS. Several mechanisms have been shown to contribute to neuronal damage in neuroinflammation, such as TRAIL-mediated apoptosis and glutamate-promoted excitotoxicity. We previously reported that the death ligand TRAIL critically contributes to irreversible CNS damage during autoimmune neuroinflammation [6], [7], [26], and demonstrated that functional blocking of TRAIL was effective in preventing caspase-dependant neuronal death in EAE [5]. Thus, as shown here *in vitro* the combination of EGCG and GA might decrease TRAIL-induced damage of neurons and oligodendrocytes in neuroinflammatory diseases.

Increase in extracellular glutamate by activated microglia during neuroinflammation can have serious consequences, as it is capable of precipitating excitotoxic cell death of neurons and oligodendrocytes by overstimulation of ionotropic glutamate receptors [27]. It has been previously shown, that targeting glutamate excitotoxicity by treatment with the AMPA/kainate antagonist NBQX resulted in substantial amelioration of EAE, increased oligodendrocyte survival and reduced axonal damage [28]. The ability of GA and EGCG in combination to effectively and synergistically reduce excitotoxicity *in vitro* might as well translate into reduced neuronal damage during EAE and MS.

Both our previous study [15] and other studies [29] have shown that neuroprotective effects of EGCG are mediated, at least in part, by reduced reactive oxygen species generation and reduction in NFκB activity.

The mechanisms underlying GA-mediated neuroprotection involve triggering T cell reactivation and inducing T cells to switch to a protective phenotype [30]. So far there is evidence suggesting that GA can also exert neuroprotection independently of T cells, which seems to be mediated through protein kinase C and BDNF [31]. Thus, our results in a T cell free system provide further evidence for a direct neuroprotective mechanism of GA in addition to T cell protective immunity.

It has been shown that multiple endogenous repair mechanisms become activated after damage to the central nervous system in the context of traumatic injury, neurodegenerative or neuroinflammatory diseases. These include repopulation of damaged tissue by precursor cells for neurons and oligodendrocytes, and remyelination, as well as sprouting and rewiring of axon collaterals, formation of new synapses and axonal regeneration. However, these endogenous self-repair mechanisms are not sufficiently effective to reverse the damage that occurs in severe or chronic disease. In MS, where demyelination and axonal damage are major hallmarks of the disease, some efforts have been made in developing regenerative therapies that further promote remyelination, but very few studies focus on promotion of axonal growth and regeneration [32], [33]. Here we showed for the first time that the immunomodulatory agent GA is able with EGCG to directly promote axon outgrowth and increased axonal thickness in primary neurons in a synergistic manner. It has been demonstrated that GA-specific T cells induce bystander effects on CNS resident cells to express beneficial cytokines, and augments the *in situ* expression of BDNF, Neutrophin 3 and Neural Growth Factor, which regulate axonal growth [13], [34]. Here we observed that GA induced the production of neurotrophins in the supernatants of the entorhinal explant cultures independent of GA-specific T cells. However, the effect of EGCG in promoting axonal growth is not mediated by neutrophins as neurotrophin levels were not increased upon combination therapy compared to GA alone.

In our previous study we demonstrated a clear disease-limiting effect of EGCG in EAE [15]. In the preventative paradigm this effect reached statistical significance after 30 days of treatment, and after 50 days in the therapeutic paradigm [15]. The results of the present study show that when combined with GA, the effects of EGCG were even more potent, as statistically significant reductions in disease scores were seen much earlier and disease severity and incidence were reduced in a synergistic manner. The effects on clinical scores were also reflected in a synergistic amelioration of CNS inflammatory pathology. Even more important for clinical application, the combination therapy of EGCG and GA also reduced severity of disease when treatment started after onset of symptoms.

An increasing body of evidence indicates that cumulative axonal loss in MS correlates with permanent clinical disability and that axonal damage begins at the earliest stages of disease [35]. There is a clear need for improved therapies that are aimed at providing neuroprotection and preventing the progression of disease to chronic disability. The synergistic immunomodulatory, neuroprotective and newly established neuroregenerative functions of EGCG and GA as well as their excellent clinical tolerability make these agents attractive therapeutic candidates for combination therapy for MS. The fact that GA and EGCG act via distinct mechanisms (immune modulation and neurotrophin production versus antioxidant and metal ion chelating actions, respectively), could explain the shown synergistic effects *in vitro* and *in vivo*. Additionally, there is evidence indicating that EGCG has the capacity to cross the blood-brain barrier [36], [37]. This would be important for exerting therapeutic benefits in the CNS in the chronic phase of MS, when acute inflammation plays less of a role in disease progression.

Thus, the combination therapy of GA and EGCG is a promising and safe approach for MS as the therapeutic effects of the already established agent GA might be enhanced by the neuroprotectant EGCG. The beneficial effects of EGCG and GA could also be relevant for other chronic neurodegenerative diseases, as Alzheimer's and Parkinson's disease; thus combination therapy with these compounds could have even broader clinical implications.
